# Pilot-Scale Cultivation of the Snow Alga *Chloromonas typhlos* in a Photobioreactor

**DOI:** 10.3389/fbioe.2022.896261

**Published:** 2022-06-09

**Authors:** Floris Schoeters, Jornt Spit, Rahmasari Nur Azizah, Sabine Van Miert

**Affiliations:** ^1^ Radius, Thomas More University of Applied Sciences, Geel, Belgium; ^2^ I-BioStat, Data Science Institute, Hasselt University, Hasselt, Belgium

**Keywords:** biomass production, greenhouse, microalgae, psychrotolerant, year-round cultivation, CO_2_ utilization, cold climate

## Abstract

The most studied and cultivated microalgae have a temperature optimum between 20 and 35°C. This temperature range hampers sustainable microalgae growth in countries with colder periods. To overcome this problem, psychrotolerant microalgae, such as the snow alga *Chloromonas typhlos*, can be cultivated during these colder periods. However, most of the research work has been carried out in the laboratory. The step between laboratory-scale and large-scale cultivation is difficult, making pilot-scale tests crucial to gather more information. Here, we presented a successful pilot-scale growth test of *C. typhlos*. Seven batch mode growth periods were compared during two longer growth tests in a photobioreactor of 350 L. We demonstrated the potential of this alga to be cultivated at colder ambient temperatures. The tests were performed during winter and springtime to compare ambient temperature and sunlight influences. The growth and CO_2_ usage were continuously monitored to calculate the productivity and CO_2_ fixation efficiency. A maximum dry weight of 1.082 g L^−1^ was achieved while a maximum growth rate and maximum daily volumetric and areal productivities of 0.105 d^−1^, 0.110 g L^−1^ d^−1^, and 2.746 g m^−2^ d^−1^, respectively, were measured. Future tests to optimize the cultivation of *C. typhlos* and production of astaxanthin, for example, will be crucial to explore the potential of biomass production of *C. typhlos* on a commercial scale.

## Introduction

Microalgae are a diverse group of single-celled eukaryotic organisms that are ubiquitously present in a wide range of habitats and conditions. They can use CO_2_ as a carbon source and sunlight as an energy source to produce organic matter through photosynthesis (Benedetti et al., 2018; Abo et al., 2019; Vale et al., 2020; Vecchi et al., 2020). Their photosynthetic efficiency can be 10 times more efficient than that of terrestrial plants, and when combined with their fast growth rates, they are a promising source of renewable feedstock for different applications (Singh and Ahluwalia, 2013; Benedetti et al., 2018; Khan et al., 2018). It is, thus, no surprise that the cultivation of microalgae has bloomed in recent years due to its vast potential (Richmond, 2000; Chisti, 2007; Singh and Ahluwalia, 2013; Benedetti et al., 2018; Khan et al., 2018). The true potential of microalgae, however, remains untapped on a larger scale. For many microalgae species, several challenges remain to reach sustainable and economically viable cultivation (Richmond, 2000; Chisti, 2007; Mata et al., 2010; Sayre, 2010; Tredici, 2010).

Currently, only a few selective microalgae are commercially cultivated (Garrido-Cardenas et al., 2018; Dolganyuk et al., 2020; Wang et al., 2021). This is often attributed to the legislative burden associated with the cultivation of novel species, for example, the European Novel Foods Regulation (Araújo et al., 2021). Next to the aforementioned, only a select few of the thousands of species are studied (Guiry, 2012; Garrido-Cardenas et al., 2018). Most of this research is carried out on the laboratory scale and often in well-controlled small volumes, making the extrapolation to larger-scale cultivation difficult (Tredici, 2010; Quinn et al., 2011; Moody et al., 2014). Furthermore, most microalgae studied or commercially cultivated are microalgae with optimal growth temperatures between 20 and 35°C (Kvíderová et al., 2017; Cheregi et al., 2019; Suzuki et al., 2019; Dolganyuk et al., 2020). Nevertheless, many countries have a colder climate, making year-round cultivation difficult unless cultures are heated during colder months. Since the production costs of microalgae are still a bottleneck in the expansion of commercialization, a year-round microalgae cultivation, without the need for excessive heating, can support sustainability and economic feasibility (Matsumoto et al., 2017; Garrido-Cardenas et al., 2018; Cheregi et al., 2019; Suzuki et al., 2019).

Cold-adapted microalgae could bridge the colder periods during year-round microalgae cultivation (Kvíderová et al., 2017; Matsumoto et al., 2017; Suzuki et al., 2019). While, cold-adapted microalgae, often referred to as snow algae, are being studied more in recent years (Remias et al., 2016; Kvíderová et al., 2017; Hoham and Remias, 2020); most studies focus on the ecology and taxonomy. A few studies describe the cultivation of snow algae in a laboratory setting, but knowledge of their cultivation on a larger scale is largely absent (Kvíderová, 2010; Kvíderová et al., 2017; Matsumoto et al., 2017). Aside from being able to grow in colder temperatures, snow algae can be a source of polyunsaturated fatty acids (PUFAs) and other valuable compounds such as astaxanthin (Varshney et al., 2015; Kvíderová et al., 2017; Cheregi et al., 2019; Sathasivam et al., 2019; Suzuki et al., 2019). Snow algae, many belonging to the *Chloromonas*–*Chlamydomonas* complex, are polyextremophiles adapted to survive in regions with harsh conditions such as low temperatures, high irradiation, and lack of nutrients and liquid water (Remias et al., 2005; Kvíderová, 2010; Kvíderová et al., 2017; Procházková et al., 2018; Hoham and Remias, 2020).

One snow alga with a potential commercial value is the red snow alga *Chloromonas typhlos* (*Chlamydomonas nivalis*). It is one of the most studied snow algae and is known to cause the phenomenon of red patches of snow (Mosser et al., 1977; Remias et al., 2005; Cvetkovska et al., 2017). This phenomenon of red snow, often called “watermelon snow”, is caused by the red pigment astaxanthin and its acid ester derivatives (Remias et al., 2005; Cvetkovska et al., 2017). However, to the best of our knowledge, no studies describe the large-scale cultivation of this alga in a photobioreactor. In order to help close the gap between laboratory experiments and large-scale cultivation of the snow alga *C. typhlos*, we performed a proof-of-concept study in which we grew *C. typhlos* in a photobioreactor installed in a minimally heated (frost-protection) greenhouse during colder periods.

## Materials and Methods

### Microalga Strain and Culture Conditions

*Chloromonas typhlos* (SAG 26.68) was purchased from SAG (Department Experimental Phycology and Culture Collection of Algae, University of Göttingen, Germany). The culture was maintained in the laboratory in a sterilized (autoclaved at 121°C for 20 min) freshwater medium based on the SAG basal medium (version 10.2008). It was kept in a 250-ml Erlenmeyer flask on an orbital shaker at 90 rpm with 70 μmol m^−2^ s^−1^ light exposure (cool-white fluorescent) in a climate-controlled room at 22°C (±0.2 SD) under a 16/8-h day/night regime. For upscaling, the cultures were transferred, respectively, to aerated 1, 2, and 40 L recipients. Ambient air was used for aeration, and no extra CO_2_ was provided during upscaling of the cultures. Cultures in the photobioreactors were grown in the same medium sterilized by filtration (0.1 μm). The freshwater medium had the following composition: 152 mg L^−1^ HNO_3_, 22 mg L^−1^ H_3_PO_4_, 148 mg L^−1^ KOH, 6.3 mg L^−1^ Fe-DTPA, 42 pg L^−1^ CuSO_4_.5H_2_O, 2.8 μg L^−1^ ZnSO_4_, 7.2 μg L^−1^ MnSO_4_, 4.3 μg L^−1^ Na_2_MoO_4_, 40 μg L^−1^ Na_2_B_4_O_7_, 0.2 g L^−1^ NaHCO_3_, and 23 mg L^−1^ MgSO_4_.7H_2_O. Furthermore, nitrogen and phosphorus were added to the culture when needed, based on the regular measurement of their concentrations, to maintain non-nutrient conditions. The medium was prepared autonomously by a central computer control unit in the greenhouse and fed to the cultures by a feed supply unit (FertiMiX 600, Hortimax).

### Horizontal Tubular Multilayer Photobioreactor

**FIGURE 1:**
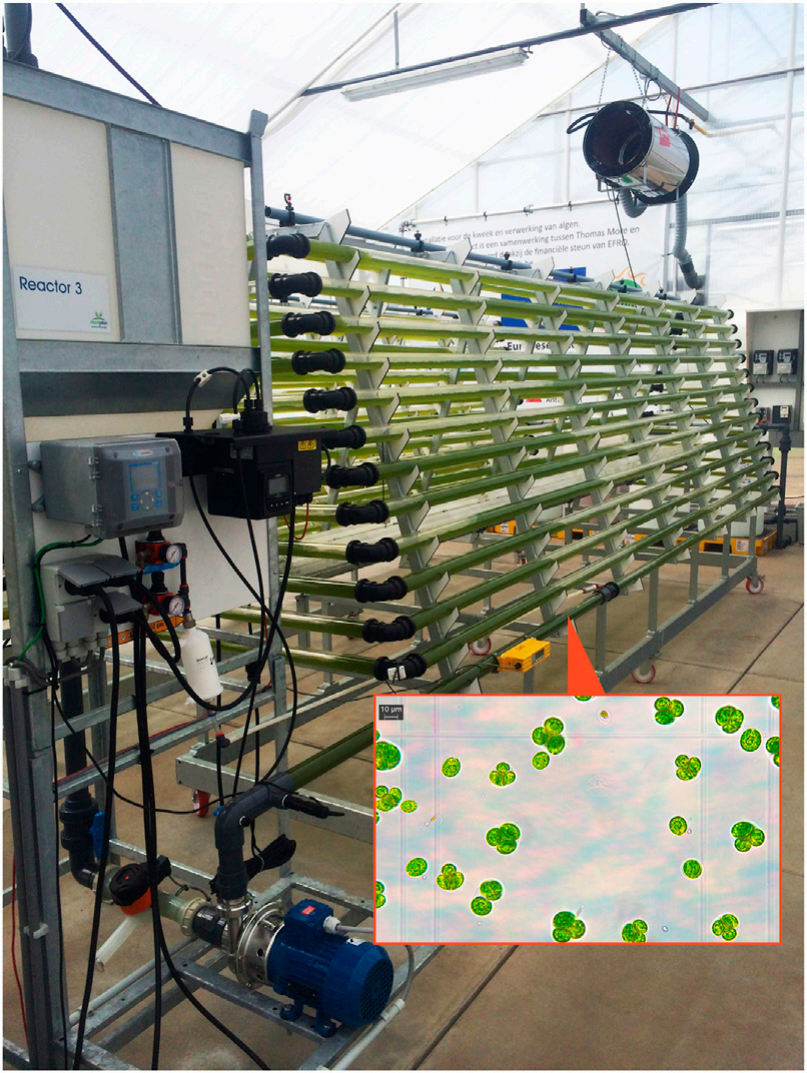
Picture of the photobioreactor used to perform the growth experiments with *C. typhlos*. The red frame shows the *C. typhlos* cells as seen throughout the experiments. The cells were always green.

A tubular multilayer photobioreactor with a volume of 350 L was used for the cultivation of *C. typhlos*. The reactor is part of the pilot plant of the EU project “Sunbuilt” and is located in a greenhouse in Geel, Belgium. The photobioreactor consists of transparent unplasticized polyvinylchloride (PVC-u) tubes with an external diameter of 5 cm. To avoid shading of lower located tubes, a triangle-like configuration of the tubes was used (Figure 1). For the inoculation of the photobioreactor, approximately 40 L of a 0.5–1 g L^−1^ *C. typhlos* culture was used.

### Modus Operandi of the Photobioreactor

*C. typhlos* was cultivated in a 350-L (V_r_) bioreactor during two growth tests ranging from March to May 2019 (growth test 1) and from December 2019 to February 2020 (growth test 2) in non-nutrient limiting conditions. To evaluate the growth of *C. typhlos* during these two tests, seven (three during the first test and four during the second test) 9-day growth periods were selected and compared. Prior to starting a 9-day growth period, *C. typhlos* was partially harvested, and the culture was refreshed with a new medium and permitted to regrow before a new 9-day growth period started. The first period at the start of growth test 2 (December 2019), however, started right from inoculation, giving a lower start concentration.

The growth was monitored continuously online with a turbidity sensor (Georg Fischer Signet 4,150 turbidimeter 0–1000 NTU) and offline by dry weight measurements. The medium was circulated by using a centrifugal pump (900 rpm), and filter-sterilized (0.1 μm) ambient air was injected into the lowest tube at a continuous rate for mixing. The working pH was set at 8, continuously measured, and maintained by the injection of CO_2_ on demand, in the airflow, with a maximum flow of 885.9 ml normal per minute. The total CO_2_ injected into the photobioreactors was measured during the growth periods (IN-FLOW mass flow meter/controller Bronkhorst, the Netherlands). The ambient temperature inside the greenhouse was monitored (Ektron III-C, Hortiplan) continuously and maintained at night at a minimal temperature of 10°C for frost protection with a gas heater (HHB-100A-230V, Holland Heaters).

When the temperature inside the greenhouse reached 23°C, foggers inside the greenhouse and sprinklers on the roof were turned on automatically to prevent an excessive rise in temperature. Photosynthetically active radiation (PAR) was measured continuously with a PAR sensor (LI-COR LI-190 R Quantum sensor) installed inside the greenhouse, on top of the reactor. A solar irradiance meter (LP02-TR pyranometer) was installed outside the greenhouse to measure total solar radiation. At a measured value of 400 W m^−2^, a sunscreen inside the greenhouse was automatically partially closed to reduce irradiation by 20–30%. The effect of additional lighting to prolong the length of daytime was studied between 10 December 2019 and 19 February 2020 by providing extra lighting (Philips TL-D 58 W 865; 504 mmol m^−2^) from 6.30 to 10.30 and 15.30 to 22.00 during 3–4 days to lengthen the day and simulate a 16/8-h day/night cycle. During the other days, the extra lighting was not switched on to see if there was a difference between days with extra lighting versus days without extra lighting. Control, logging, and steering were carried out automatically by computer (MultiMate series III, Hortiplan).

### Growth Determination

The algal growth in the bioreactor was monitored online every 30 min with a turbidity meter. The measured turbidity was correlated with a dry weight value based on a correlation described previously (Thoré et al., 2021). In addition to the continuous monitoring by turbidity, the dry weight (g L^−1^) was determined at the start (C_s_) and end (C_e_) of each batch and at random moments during the growth periods. For this, samples (5 ml/sample) were filtered over pre-dried and pre-weighed Whatman GF/C glass microfiber filter papers (0.45 µm) and washed. After drying for 24 h at 70°C, the samples were cooled in a desiccator before weighing.

### Calculations of Volumetric and Areal Productivities, Specific Growth Rate, CO_2_ Fixation, Influence of Total PAR, and CO_2_ Utilization Efficiency

The total volumetric biomass productivity P_v_ (dry g L^−1^ d^−1^) for the batch mode was calculated by the formula:

$${\text{P}}_{\text{v}}=\left({\text{C}}_{\text{e}}-{\text{C}}_{\text{s}}\right)/{\text{t}}_{\text{d}},$$

with C_s_ and C_e,_ the start and end biomass (g L^−1^), respectively, and t_d_ was the cultivation period (9 days for total and 1 day for (maximum) daily volumetric biomass productivity). The total areal biomass productivity P_a_ (dry g m^−2^ d^−1^) was calculated as follows:

$${\text{P}}_{\text{a}}=350\left({\text{C}}_{\text{e }}-{\text{C}}_{\text{s}}\right)/14{\text{t}}_{\text{d}},$$

with 350 L being the volume of the reactor, t_d_ being 9 days for the total or 1 day for the (maximum) daily biomass areal productivity, and 14 m^2^ being the total area covered by the reactor.

The specific growth rate was calculated over the 9-day period:

$$µ=\text{ln}\left({\text{N}}_{\text{e }}/{\text{N}}_{\text{s}}\right)/{\text{t}}_{\text{d}},$$

with N_e_ being the biomass at the end and N_s_ being the biomass at the start of the 9-day period and t_d_ being 9 days.

To know the daily yield on light (P_L_), the daily areal productivity (P_a_) was divided by the total PAR (PAR_T_) received for that specific day, resulting in P_L_:

$${\text{P}}_{\text{L}}={\text{P}}_{\text{a}}/{\text{PAR}}_{\text{T}}.$$

The total CO_2_ injected was measured during each 9-day period by using a mass flow meter (IN-FLOW mass flow meter/controller Bronkhorst, the Netherlands). The theoretical total CO_2_ fixation (CO_2th_, g) by the algae for each batch was calculated as follows:

$${\text{CO}}_{2\text{th}}=0.5\left({\text{C}}_{\text{e}}-{\text{C}}_{\text{s}}\right){\text{V}}_{\text{r}}\left(\frac{{\text{m}}_{{\text{CO}}_{2}}}{{\text{m}}_{\text{C}}}\right),$$

with ${\text{m}}_{{\text{CO}}_{2}}{\text{ and m}}_{\text{C}}$ being the molar masses of CO_2_ and C, respectively, V_r_ the volume of the reactor, and 0.5 being the average percentage of the carbon content in the biomass (Mirón et al., 2003; Tang et al., 2011). Higher or lower carbon content is nonetheless possible (Mandalam and Palsson, 1998; Patil et al., 2012; Rendón-Castrillón et al., 2021) which might lead to significant differences (Adamczyk et al., 2016).

The CO_2_ fixation rate CO_2fr_ was calculated as $1.833{\text{P}}_{\text{v}},$ with 1.833 derived from multiplying the average carbon content (50%) per unit of biomass with the CO_2_ carbon ratio (44/12).

To have an idea about the CO_2_ uptake efficiency, the calculated CO_2th_ was divided by the total CO_2_ (CO_2t_, g) injected (influent CO_2_) and multiplied by 100 to know the utilization efficiency percentage (U_%_):

$${\text{U}}_{\text{\%}}=100{\text{CO}}_{2\text{th}}/{\text{CO}}_{2\text{t}}.$$

The aforementioned formula is a simplified version of the formula: $\frac{{\text{Influent of CO}}_{2}-{\text{effluent CO}}_{2}}{{\text{influent CO}}_{2}}100$ (Klinthong et al., 2015), in which the numerator is replaced by the theoretical total CO_2_ fixation since the photobioreactor had no measurement of CO_2_ in the outgoing gases. The total loss of CO_2_ (CO_2tL_) was calculated by subtracting CO_2th_ from CO_2t_:

$${\text{CO}}_{2\text{tL}}={\text{CO}}_{2\text{t}}-{\text{CO}}_{2\text{th}}.$$

### Statistical Analysis

To compare the differences in temperatures, received PAR, and daily biomass production between the seven batches, a one-way analysis of variance (ANOVA) with *post hoc* Tukey HSD with a significance level of 0.05 was performed. Normality was tested with a Shapiro–Wilk test. Data are given as mean ± SD.

## Results and Discussion

### Growth and Biomass Productivity

**FIGURE 2:**
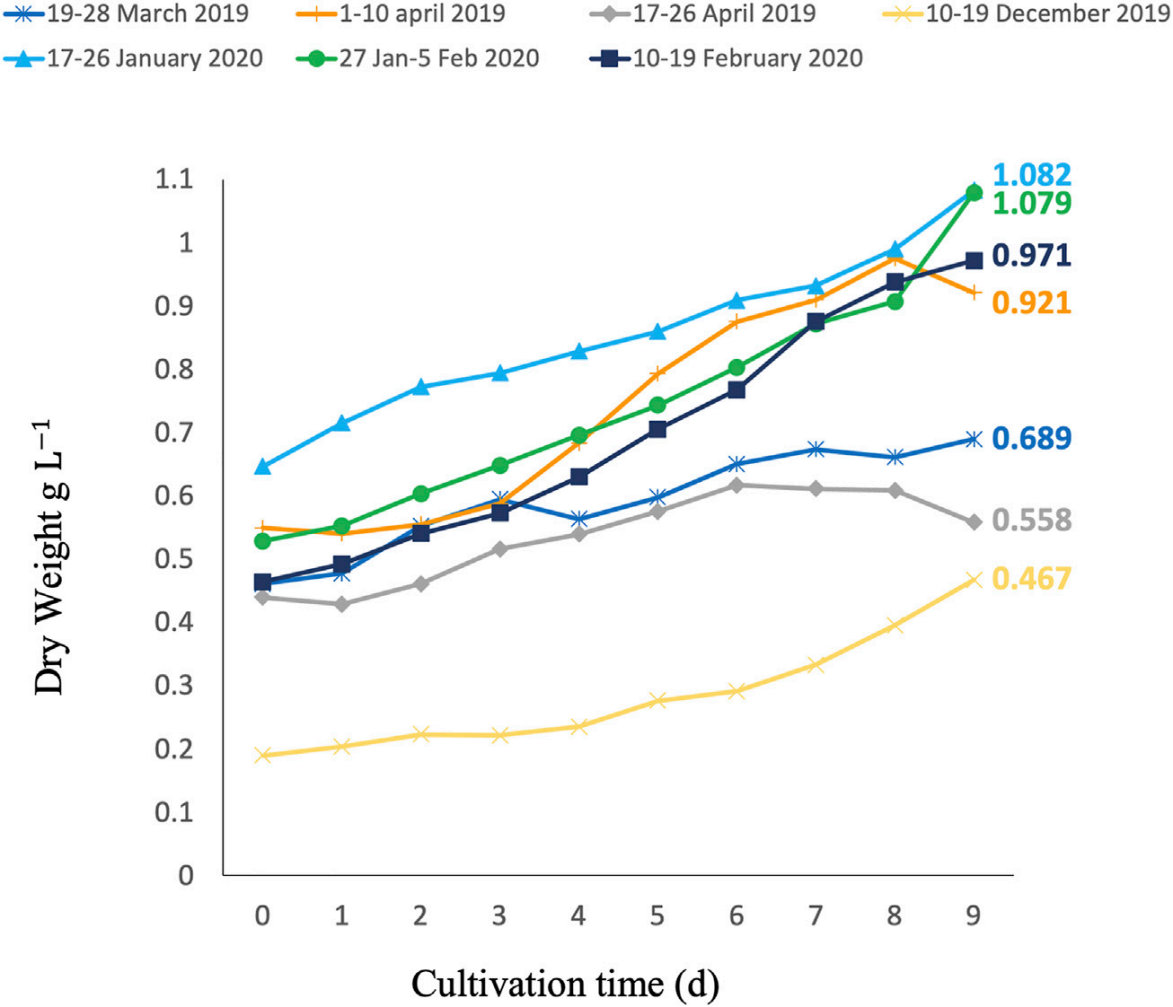
Growth curves of *C. typhlos* during seven different 9-day batch growth tests in a 350-L photobioreactor. The end concentration (C_e_) of each growth period is depicted next to the respective lines in g L^−1^.

To analyze the 9-day periods, the daily productivities were compared, and a difference was found (*p* < 0.05): the period 17–26 April was significantly different from periods 17–26 January 2020 and 27 January–5 February 2020, while the period 27 January–5 February 2020 was also significantly different from the period 10–19 December 2019. To further assess the biomass obtained during the cultivation of *C. typhlos* in the seven 9-day periods, we looked at the specific growth rate and total and maximum volumetric and areal biomass productivities. Table 1 summarizes these results. The highest growth rate was achieved during the 10–19 December period, which was five times higher than the 17–26 April period. Figure 2 shows that the growth halted after 6 days in the 17–26 April period, leading to a lower growth rate. The highest total volumetric biomass productivity (0.067 g L^−1^ d^−1^) was obtained during the period 27 January–5 February 2020. While a maximum biomass productivity of 0.110 g L^−1^ d^−1^ was obtained during the period 1–10 April 2019, the total volumetric productivity (0.046 g L^−1^ d^−1^) was lower than that of three other periods during the winter (Table 1).

**TABLE 1:** Volumetric and areal biomass productivities in dry weight of *C. typhlos* grown in batch for 9-day periods in a 350-L horizontal tubular reactor and the specific growth rates are shown. The specific growth rate (µ) is calculated over the 9-day period. The total volumetric (P_v_) and areal (P_a_) productivities are calculated over the 9-day period by dividing the total dry weight produced by 9. The maximum volumetric (Max P_v_) or areal (Max P_a_) productivity is the maximum value obtained for the daily productivity during that 9-day period.

Period	µ	P_v_	Max P_v_	P_a_	Max P_a_
	d^−1^	g L^−1^ d^−1^	g L^−1^ d^−1^	g m^−2^ d^−1^	g m^−2^ d^−1^
19–28 March 2019	0.050	0.028	0.075	0.694	1.871
1–10 April 2019	0.066	0.046	0.110	1.147	2.746
17–26 April 2019^a^	0.020	0.010	0.055	0.252	1.384
10–19 December 2019^b^	0.105	0.032	0.063	0.791	1.561
17–26 January 2020^b^	0.072	0.057	0.069	1.431	1.717
27 January–5 February 2020^b^	0.091	0.067	0.069	1.680	1.720
10–19 February 2020^b^	0.091	0.060	0.108	1.508	2.695
Mean	0.0707 ± 0.029	0.043 ± 0.021	0.078 ± 0.021	1.072 ± 0.514	1.956 ± 0.543

a Growth during this period was comparable with other periods during the first 4–5 days but halted afterward. This is most likely due to 5 consecutive days of ambient temperatures over 30°C. See text for more details.

b Denotes periods in which artificial lighting was provided; see text for more details.

**FIGURE 3:**
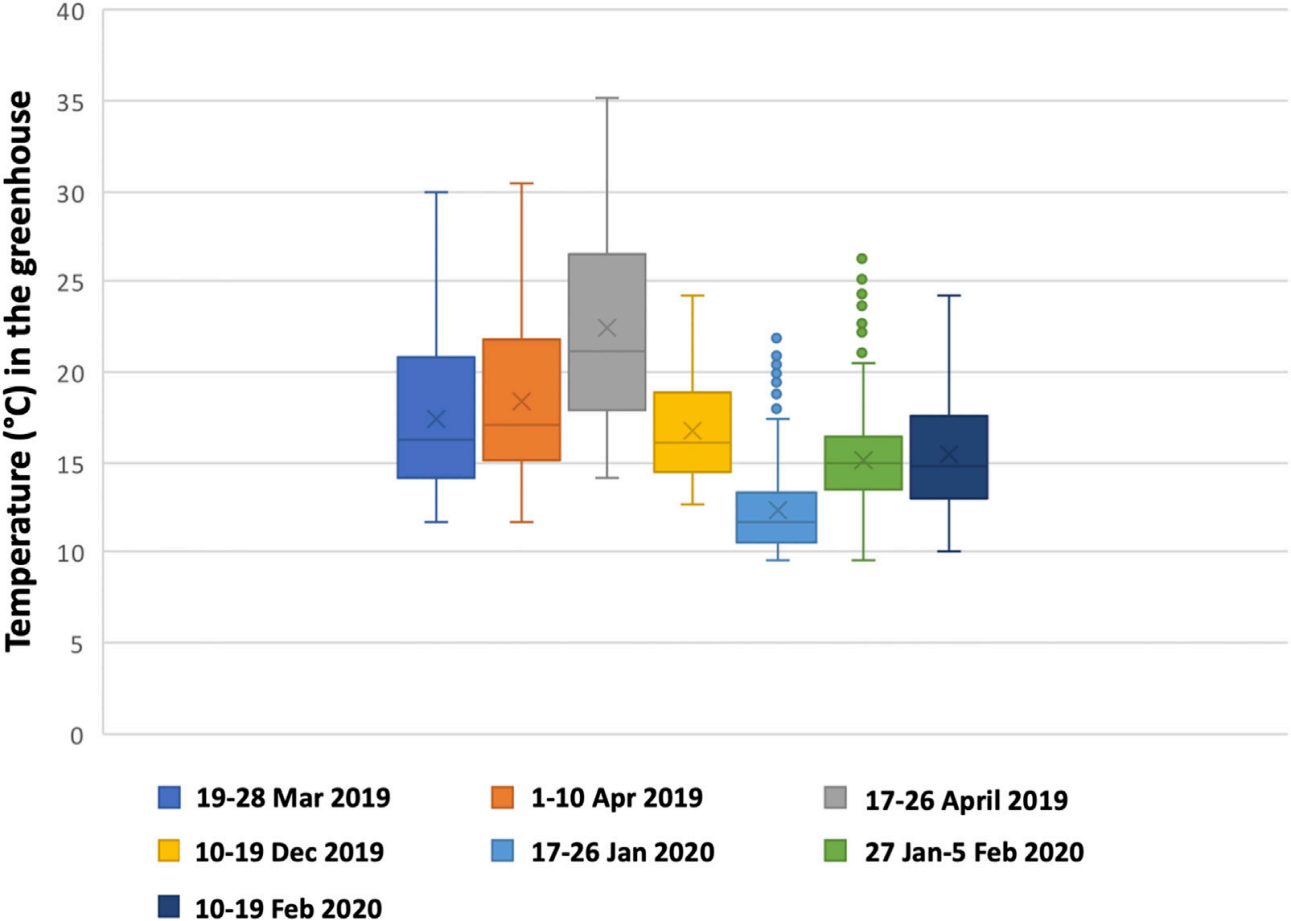
Box plot of the temperature for each period. Outliers are shown by dots above the maximum. See also Table 2.

A potential explanation for the lower total volumetric productivity during the 1–10 April period could be that a maximum ambient temperature of 30.5°C was reached inside the greenhouse on 7 April 2019, causing temperature stress on the cells and leading to a decline in growth. This can be seen in Figure 2 between days 8 and 9. A similar effect could be observed from day 6 during the period 17–26 April 2019. During this period, the lowest total volumetric biomass productivity (0.010 g L^−1^ d^−1^) and growth rate (0.020 d^−1^) were obtained. This growth period had the highest average temperature (22.5°C), the highest maximum temperature (35.2°C), and several (5) consecutive days with temperatures above 30°C (Figure 3). A stress response at a temperature around 30°C is in line with what others have reported (Teoh et al., 2013; Lukeš et al., 2014). Contrary to Teoh et al. (2013) and Lukeš et al. (2014), we measured the ambient temperature in the greenhouse rather than the culture temperature itself. Since multiple parameters can influence the culture temperature and large differences between ambient and culture temperature are possible (Tredici and Materassi, 1992; González-Camejo et al., 2019), future laboratory and pilot-scale tests that measure both the ambient and culture temperatures are required to fully understand the temperature impact on *C. typhlos*.

Productivities and growth rates for the *Chloromonas* species were lower than the literature values: over the nine batches average volumetric and areal productivities of 0.043 ± 0.021 g L^−1^ d^−1^ and 1.072 ± 0.514 g m^−2^ d^−1^, respectively, were obtained, while growth rates varied between 0.020 and 0.105 d^−1^. However, a comparison is difficult to obtain due to insufficient information or the different cultivation techniques used (Teoh et al., 2013; Idrissi Abdelkhalek et al., 2016; Hulatt et al., 2017; Onuma et al., 2018; Morales-Sánchez et al., 2020; Peng et al., 2021). Compared to commercially cultivated algae, the production and growth rate is lower and needs to be improved to be competitive (de Vree et al., 2015; Barka and Blecker, 2016; Benedetti et al., 2018; Darvehei et al., 2018; Metsoviti et al., 2019).

Aside from temperature stress, algae, in general, react to light stress (Minhas et al., 2016; Shi et al., 2020; Zheng et al., 2020), although *C. typhlos* has mechanisms; for example, the production of astaxanthin to cope with high light intensities (Gorton et al., 2007; Remias et al., 2010; Zheng et al., 2020). To estimate the influence of total PAR on the growth of *C. typhlos*, the total averaged PAR was measured. Table 2 shows the average PAR received during the periods, the average daily yield on light (P_L_), average temperatures, and whether they differ significantly between growth periods.

**TABLE 2:** Average total PAR, average daily yield in light (P_L_) for each growth period, and greenhouse temperatures measured during the batch tests. A significance between groups is also shown with *p* < 0.05. The identifier is used to show the significant differences between periods.

Period (ID)	Average total PAR	Significant versus	Average PL	Significant versus	Average temperature	Significant versus
	**Mmol m^−2^**		**g μmol^−1^ d^−1^**		**°C**	
19–28 March 2019 (A)	3,074 ± 365	C	0.22 ± 20	/	17.5 ± 0.9	C, E, and F
1–10 April 2019 (B)	3,446 ± 467	G	0.31 ± 29	/	18.4 ± 1.4	C, E, F, and G
17–26 April 2019 (C)	4,283 ± 509	A, D, E, and F	0.1 ± 0.12	E, F	22.5 ± 2.5	A, B, D, E, F, and G
10–19 December 2019^a^ (D)	2,709 ± 449	C and G	0.26 ± 0.24	/	16.7 ± 1.8	C and E
17–26 January 2020^a^ (E)	2,888 ± 770	C and G	0.39 ± 0.15	C	12.4 ± 1.4	A, B, C, D, F, and G
27 January–5 February 2020^a^ (F)	3,379 ± 925	C and G	0.40 ± 0.25	C	15.1 ± 1.0	A, B, C, and E
10–19 February 2020^a^ (G)	5,039 ± 727	B, D, E, and G	0.25 ± 0.16	/	15.5 ± 1.5	B, C, and E

a During these periods artificial light was provided. The artificial light is included in the average totals depicted here.

During the 10–19 February period, the total PAR was higher than the spring growth periods. There was a higher total PAR during the February 2020 period due to a few exceptional sunny days throughout this period and the extra artificial lighting. In addition, the sun screens in the greenhouse did not partly close since the minimal closing limit of 400 W m^−2^ was most often not reached, contrary to the spring periods. Table 2 shows that the growth period 17–26 of January had the lowest temperature and second lowest total PAR light, yet the total volumetric and areal productivities were above the averages of the growth periods (see Table 1). This shows that even at lower temperatures and sunlight, *C. typhlos* can still be cultivated at comparable productivities to higher temperature and sunlight conditions.

The total PAR received differed significantly between the growth periods. To normalize the growth for PAR received, we looked at the yield per light. Only period 17–26 April 2019 was significantly different from periods 17–26 January 2020 and 27 January–5 February 2020. The time period of 16–26 April 2019 was also significantly different from all other periods in regard to the temperatures reached (Table 2). The period 17–26 April 2019 was also different than most other growth periods for the total light received (only periods 1–10 April 2019 and 10–19 February 2020 were similar). This shows the influence of higher temperatures (>30°C) on the growth of *C. typhlos*. Table 2 also shows that at lower temperatures, *C. typhlos* still had a high yield in light.

Like many cold adapted algae, *C. typhlos* is a potential source of lipids and carotenoids produced at lower temperatures and high light intensities as a mechanism to protect the cells (Gorton et al., 2007; Remias et al., 2010; Liu and Nakamura, 2019; Hoham and Remias, 2020; Shi et al., 2020; Peng et al., 2021). If *C. typhlos* can produce sufficient biomass and valuable products such as astaxanthin at a low temperature with high light intensities, it can be a sustainable approach to cultivate microalgae during colder periods without excessive heating.

During our growth experiments, *C. typhlos* cells were green (Figure 1). We did not notice the typical red color indicative of astaxanthin. All experiments were performed in non-nutrient limiting batches, and no attempts were made during these experiments to stress the cells specifically to produce astaxanthin and color red. As no analysis was performed on the astaxanthin content, we cannot rule out that it was produced at levels too low to give the cells their typical red color. For future work, it will be vital to characterize the specific parameters, such as low nitrogen, that produce red *C. typhlos* cells as astaxanthin is an important and valuable carotenoid (Leya et al., 2009; Han et al., 2013; Sathasivam et al., 2019).

### Turbidity Measurements to Monitor Growth and Influence of (Artificial) Light

**FIGURE 4:**
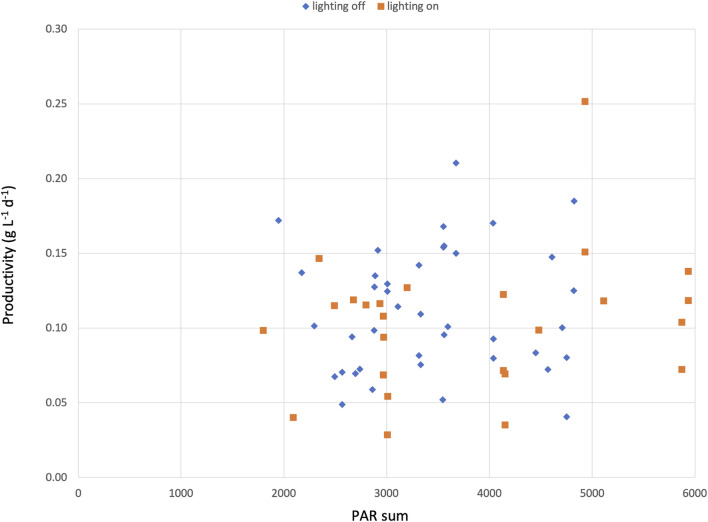
Daily productivity of *C. typhlos* (g L^−1^ d^−1^) and PAR sum of each specific day during the two growth periods. Each square represents 1 day.

During the winter period, artificial lights were turned on for several days in each period to simulate a 16/8 day-night regime. These days were then compared to days without artificial light. However, no significant difference in biomass productivity was found between the days with artificial light versus the days without (Figure 4). Several reasons might explain why the extra lighting had no effect. One explanation could be that our 9-day test periods were too short to detect a difference. A second reason could be the high variance of received sunlight between the days. The PAR sum from the sunlight ranged from 1,588 to 5,431 mmol m^−2^. On days with the addition of artificial light, the PAR sum increased by 10–31%; however, due to the high variance, the effect was limited. A third reason could be the use of insufficient or non-specific lighting. The reactors were constructed in 2014 and equipped with fluorescent lamps (6500 K) that only provided minimal extra lighting during the dark period. However, in recent years, more attention has been given to the use of modern LED lamps with a specific light spectrum to optimize the growth of microalgae (Glemser et al., 2016); using better lights might therefore have a significant impact on growth. A last final reason could be the placing of the lights. They were fixed underneath the PBR tubes and only reached a small area of the total PBR tubes. Placing LED lamps closer to each individual tube could increase the effects substantially.

**FIGURE 5:**
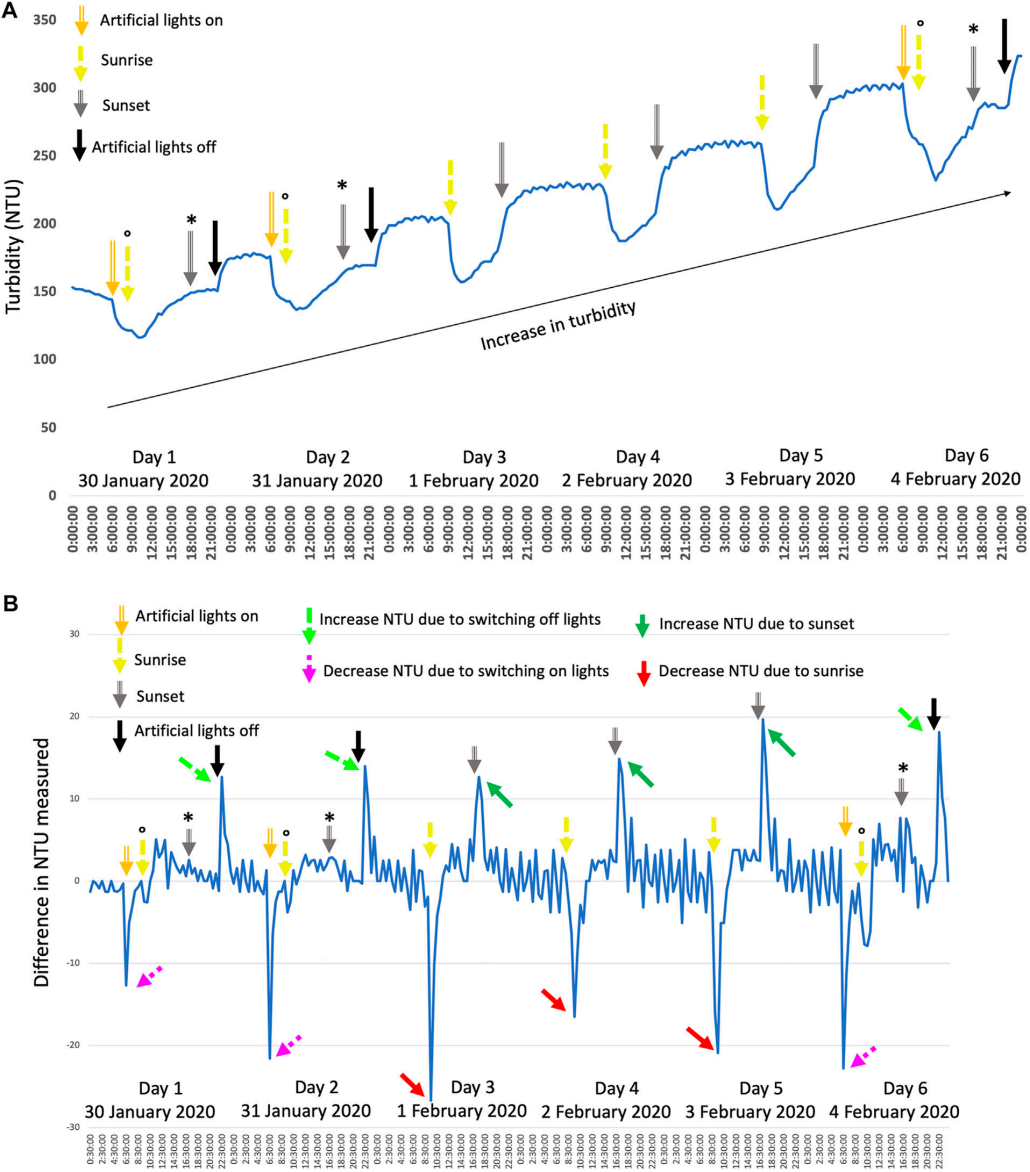
**(A)** shows the typical growth pattern of *C. typhlos* observed over several days. A representative period during one of the winter periods is shown here with artificial lights being used to extend the day. Artificial lights were kept on between 6.30–10.30 and 15.30–22.00, although slight variances were possible depending on the real sunset and sunrise times of the specific days. **(B)** shows the difference in the NTU value calculated for every 30 min (NTUx+30min—NTUx) plotted over time. Arrows indicate specific time points or substantial increases/decreases. The symbol ° denotes the artificial light switching off at sunrise, while the symbol * denotes the artificial lights switching on at sunset. See text for more details.

Figure 5A shows a typical growth pattern based on turbidity measurements (NTU). A general increase in turbidity can be seen over the course of several days (the black arrow in Figure 5A), indicating an increase in cells and biomass. When looking at a 24-h period, without artificial light added on days 3, 4, and 5, a typical pattern can be observed, pointing toward a diurnal rhythm. The cell cycle of photosynthetic microalgae is influenced by a day-night regime in which cell growth (G1) takes place during the light phase (daytime), and the reproductive, cell division phase (S/M) occurs during the dark period (nighttime) (Cross and Umen, 2015; Ivanov et al., 2019; Zou and Bozhkov, 2021). The NTU profile (Figure 5A) can be linked to this diurnal rhythm. The steady increase in NTU a few hours after sunrise to sunset follows the cell growth: the enlargement of the cells and increase in dry weight (Lien and Knutsen, 1979; Spudich and Sager, 1980; Ostgaard and Jensen, 1982; Bišová and Zachleder, 2014; de Winter et al., 2017b). After sunset and during the night, the NTU fluctuated little, which can be linked to the cells no longer growing but dividing within the parental cell (Harper, 1999; Cross and Umen, 2015; Ivanov et al., 2019; Zou and Bozhkov, 2021). Throughout warmer periods, the NTU decreased more overnight, which can be linked to the loss of biomass during the night. During warmer nights, the respiration rates can be higher than on colder nights (De-Luca et al., 2019; Masojídek et al., 2021), leading to more biomass loss and potentially explaining the more pronounced decrease in NTU during warmer nights.

This nightly biomass loss is often attributed to respiration using compounds like carbohydrates during the night (Ogbonna and Tanaka, 1996; Michels et al., 2014; Edmundson and Huesemann, 2015; Åkerström et al., 2016). Next to the aforementioned, general pattern, a rapid and substantial drop or increase in turbidity, respectively, at sunrise and sunset, is seen. To highlight this, Figure 5B shows the difference in NTU between each half-hour registered. The drop in turbidity at sunrise is indicated with red arrows, while the increase at sunset is visualized with green arrows. A rapid decrease or increase points toward a strong and swift reaction of the algae to the presence or absence of light. The NTU is monitored with a nephelometric sensor that uses white light (400–700 nm) that can readably be used by microalgae (Carvalho et al., 2006; de Mooij et al., 2016). Potentially, *C. typhlos* is able to activate and upregulate its photosynthetic system very fast, leading to a substantial increase in absorption of light while reducing the scattered light and causing a rapid and substantial drop in turbidity (Thorne and Nannestad, 1959; Kourti, 2000; Gregory, 2009). For extremophiles, a fast activation or deactivation of their metabolism can be crucial to cope with harsh conditions (Harel et al., 2004; Peng et al., 2021).

As daughter cells are released from the parental cell at the end of the dark period (Mihara and Hase, 1971; Vítová and Zachleder, 2005; Harris et al., 2009; Bišová and Zachleder, 2014; Zou and Bozhkov, 2021), although it can extend into the light period (Eschbach et al., 2005), many new cells are present and able to influence the turbidity measurements when their photosynthetic apparatus is turned on at sunrise. See also Figure 1 for daughter cells still inside the parental cell after multiple fission. A similar explanation can be given for the substantial rise in NTU at sunset. As the cells switch their metabolism from the light phase to the dark phase, less light is absorbed, thereby increasing the scattering of light and giving a higher NTU value at sunset and during the night.

When artificial light was provided (days 1, 2, and 6), a similar pattern could be observed. A steep decrease in turbidity was observed when the artificial light was turned on prior to sunset (purple dashed arrows), and an increase was seen when turning off the artificial light after sunset (light green dashed arrow; Figure 5B). Figure 5A shows that the effect of the artificial light was similar but not exactly the same. The steady increase of turbidity (NTU) during the growth phase was lower when only artificial light was present as compared to sunlight (plateau between gray and black arrow). However, the substantial increase in turbidity was similar when turning off the artificial light compared to at sunset (Figure 5B, dashed light green arrows). Figure 5B also shows that after turning on the artificial light before sunrise a second, lower drop in turbidity was observable at sunrise (yellow arrows with the symbol “o” on top). Although no significant effect of artificial light was detected on the biomass production of *C. typhlos*, artificial light did, however, have a noticeable impact on the growth pattern of *C. typhlos*.

It is possible to use a continuous turbidity measurement in combination with the correlation between turbidity and dry weight to track the dry weight evolution (Thoré et al., 2021). Our results, however, also indicate that caution is needed when interpreting and linking turbidity measurements with the dry weight of the microalga cultivated. However, the growth (turbidity) pattern of *C. typhlos* was also different compared to other non-snow algae tested. Both *P. purpureum* and *N. gaditana* showed a substantial decrease in turbidity during the night (unpublished data) but no substantial decrease or increase respectively at sunrise and sunset. This could potentially indicate a different and faster response of snow algae to a night and day cycle. Like biomass loss overnight, the influence of the day/night cycles of microalgae and their effect on the cells, cell division, and biomass are still understudied and can vary between algae (Edmundson and Huesemann, 2015; de Winter et al., 2017a; León-Saiki et al., 2018). To optimize microalgae cultivation, the nightly biomass loss and circadian rhythm can be important factors to establish the optimal time of harvesting. Future studies should address this.

### CO_2_ Fixation and Utilization Efficiency

To investigate the CO_2_ fixation and utilization efficiency, the total CO_2_ injected into the photobioreactor was monitored. Using the total biomass produced during each period, the efficiency of CO_2_ uptake could be calculated (Table 3).

**TABLE 3:** Overview of the total CO_2_ injected during each batch growth test, pH was set at 8, and CO_2_ was injected on demand to maintain a steady pH. The two periods in bold show the highest CO_2_ utilization efficiency.

Period	Average pH	Average temperature	$\left(C_{e}-C_{s}\right)$	CO_2t_	CO_2th_	CO_2tL_	U%	CO_2fr_
			g L^−1^	g	g	g	%	g L^−1^ d^−1^
19–28 March 2019	8.21 ± 0.49	17.5 ± 0.9	0.250	5,390	160	5,220	2.97	0.05
1–10 April 2019	8.00 ± 0.25	18.4 ± 1.4	0.413	7,620	265	7,355	3.48	0.08
17–26 April 2019	7.93 ± 0.18	22.5 ± 2.5	0.091	2,430	58	2,372	2.40	0.02
**10–19 December 2019**	7.86 ± 0.25	16.7 ± 1.8	0.285	610	182	428	**29.97**	0.06
**17–26 January 2020**	7.80 ± 0.30	12.4 ± 1.4	0.515	860	330	530	**38.42**	0.11
27 January–5 February 2020	7.88 ± 0.24	15.1 ± 1.0	0.605	4,260	388	3,872	9.11	0.12
10–19 February 2020	7.96 ± 0.39	15.5 ± 1.5	0.543	3,980	348	3,632	8.75	0.11

During the periods 10–19 December 2019 and 17–26 January 2020, the CO_2_ utilization efficiency was the highest at 29.97 and 38.42%, respectively. The other periods achieved lower utilization efficiencies with percentages between 2.4 and 9.11%. The exact reasons for this difference are unclear as many factors can influence the CO_2_ utilization efficiency (Ryu et al., 2009; Daneshvar et al., 2022). Both periods have the lowest total PAR received (2,379 ± 345 and 2,604 ± 791 mmol/m^2^); however, they do not differ significantly from the periods 19–28 March 2019 and 10–19 February (Table 2).

A second observation is that the maximum greenhouse temperatures reached during these two periods were also the two lowest registered maximum temperatures, but the average temperature of the period 10–19 December was higher than that of three other periods (Table 2). During our tests, the worst efficiencies were seen during the three warmest periods (19–28 March 2019, 17.46 ± 0.9°C; 1–10 April 2019, 18.4 ± 1.4°C; and 17–26 April 2019, 22.4 ± 2.5°C). This is in line with the lower solubility of CO_2_ at higher temperatures (Singh and Singh, 2014). While we observed a wide range of CO_2_ fixation rates and utilization efficiency, they are in accord with what others have already found for other algal species (Tang et al., 2011; Ketheesan and Nirmalakhandan, 2012; Lam et al., 2012). In laboratory conditions and smaller working volumes, one can easily maintain specific parameters such as total PAR, pH, temperature, or a day/night period; however, it is less possible in a greenhouse pilot plant or outdoors. In order to truly find a correlation between different parameters and CO_2_ utilization to improve the utilization efficiency, more pilot plant studies are needed. CO_2_ utilization efficiency can be very low and is influenced by many parameters such as the temperature, pH, and growth rate, making the design of a reactor with optimal gas exchange expensive and very difficult (Carvalho et al., 2006; Klinthong et al., 2015; Fu et al., 2019), if not impossible. In different (seasonal) weather conditions outdoors or in a greenhouse, it can particularly be arduous. Thus, a more interesting approach for improved sustainability might be to capture the CO_2_ in the effluent gas and reuse it for the influent, lowering the total CO_2_ lost into the air.

## Conclusion

The psychrotolerant microalga *C. typhlos* was successfully cultivated in a greenhouse in a photobioreactor with a working volume of 350 L during several batch periods, ranging from winter to spring. A maximum dry weight and growth rate of 1.082 g L^−1^ and 0.105 d^−1^, respectively, were achieved while maximum volumetric and areal productivities of 0.110 g L^−1^ d^−1^ and 2.746 g m^−2^ d^−1^, respectively, were measured. Being one of the first pilot-scale cultivation tests of a snow alga in a photobioreactor, it is difficult to compare with known data from snow algae. One critical factor, negatively influencing the growth, was shown to be ambient temperatures of >30°C. Lower temperatures (11–15°C), however, had no detrimental effect on the growth. Due to the distinct growth pattern influenced by sunset or artificial light, it will be crucial to determine how to use artificial lights to find the optimal light/dark regime. While these experiments were carried out at a set pH of 8, future tests at different pH values will be crucial to determine the optimal pH and CO_2_ uptake efficiency. Future tests to optimize the cultivation and production of *C. typhlos* biomass are, thus, crucial to estimate its commercial value. Furthermore, the production of astaxanthin or PUFAs has to be studied to fully explore the potential of *C. typhlos*.

## Data Availability

The raw data supporting the conclusions of this article will be made available by the authors, without undue reservation.
