# Correction: Pilot-scale cultivation of the snow alga *Chloromonas typhlos* in a photobioreactor

**DOI:** 10.3389/fbioe.2025.1697072

**Published:** 2025-10-01

**Authors:** Floris Schoeters, Jornt Spit, Rahmasari Nur Azizah, Sabine Van Miert

**Affiliations:** ^1^ Radius, Thomas More University of Applied Sciences, Geel, Belgium; ^2^ I-BioStat, Data Science Institute, Hasselt University, Hasselt, Belgium

**Keywords:** biomass production, greenhouse, microalgae, psychrotolerant, year-round cultivation, CO_2_ utilization, cold climate

**Equation** in Materials and Methods, Calculations of Volumetric and Areal Productivities, Specific Growth Rate, CO_2_ Fixation, Influence of Total PAR, and CO_2_ Utilization Efficiency was erroneously given as [ $µ=\ln\left(N_{e}–\;N_{s}\right)/t_{d}$ ]. The correct equation is [ $µ=\ln\left(N_{e}/N_{s}\right)/t_{d}$ ].

The original article has been updated.

